# Overall Survival Prediction of Advanced Cancer Patients by Selection of the Most Significant Baseline Serum Biomarker Combination

**DOI:** 10.3389/pore.2022.1610004

**Published:** 2022-01-31

**Authors:** Daniel Deme, Sandor Kovacs, Andras Telekes

**Affiliations:** ^1^ Department of Medical Oncology, Szent Lázár County Hospital, Salgótarján, Hungary; ^2^ Department of Economical and Financial Mathematics, University of Debrecen, Debrecen, Hungary

**Keywords:** overall survival, advanced cancer, serum biomarkers, prognostic importance, CRP, albumin, PLR

## Abstract

**Introduction:** Consistent association between elevated baseline serum values and C-reactive protein (CRP), cross-linked fibrin degradation products (D-dimer), lactate dehydrogenase (LDH), decreased baseline serum albumin, absolute lymphocyte count to absolute monocyte count ratio (LMR), elevated absolute neutrophil count to absolute lymphocyte count ratio (NLR), elevated platelet count to absolute lymphocyte count ratio (PLR), and between some combinations of these biomarkers and the short overall survival of patients with malignant diseases has already been reported. These biomarkers are independent prognostic factors for cancer. Here, the most significant biomarker combination of these values was searched and studied in real-life advanced cancer patients of a single center.

**Methods:** The authors retrospectively analyzed the association of the aforementioned biomarkers and their combination and OS of 75 consecutive cancer patients with locally advanced, recurrent, or metastatic diseases. Validated cut-off determination was used.

**Results:** CRP, albumin, and PLR showed marked association with OS. Cut-off values for significant shorter OS were 30.65 mg/L (*p* < 0.001), 44.35 g/L (*p* < 0.001), and 168.20 (*p* < 0.001), respectively. Based on assessed biomarker cut-offs, four patient groups were created to determine whether biomarker values were out of range (ORV) compared to cut-off: 1) No ORV biomarkers (n = 24; OS = 26.07 months); 2) one ORV biomarker (n = 21; OS = 13.50 months); 3) two ORV biomarkers (n = 20; OS = 7.97 months), and 4) three ORV biomarkers (n = 10; OS = 3.91 months). Significant differences in OS were detected between the groups: For 1. vs. 2. hazard ratio (HR) = 3.0 (95% CI: 1.5–6.2), *p* = 0.003; for 1. vs. 3. HR = 4.1 (95% CI: 2.0–8.3), *p* < 0.001; and for 1. vs. 4. HR = 10.2 (95% CI: 4.2–24.6), *p* < 0.001.

**Conclusion:** Based on our analysis, we can confirm that the complex monitoring of CRP, albumin, and PLR would provide a good estimation of OS. Large scale prospective studies are warranted to explore this and other useful combinations of prognostic biomarkers and their relationship to the well-established prognostic systems in real-life.

## Introduction

Some routinely measured laboratory analyte baselines have been shown to have prognostic importance in malignant diseases. Both prospective and retrospective studies and also meta-analyses have described the poor prognostic role of elevated baseline C-reactive protein (CRP) (1–4), cross-linked fibrin degradation products (D-dimer) (5–8), lactate dehydrogenase (LDH) (9–12), and decreased albumin (13–15) in cancer. Deme and Telekes have also reviewed the value of elevated CRP (16), D-dimer (17), LDH (18), and decreased albumin (19) for poor outcomes of cancer patients. Decreased lymphocyte to monocyte ratio (LMR) is a factor for adverse prognosis in several cancers (20–24). Based on a large scale (25) and further smaller meta-analyses (26–33), a high absolute neutrophil count to absolute lymphocyte count ratio (NLR) has also been associated with short overall survival (OS) in many solid malignant diseases. Elevated platelet count to absolute lymphocyte count ratio (PLR) was also shown to be an adverse prognostic factor in various cancers (34–45).

Here, we evaluated the associations of baseline CRP, D-dimer, LDH, albumin, LMR, NLR, and PLR with the outcome of 75 consecutive patients with advanced cancer suitable for anticancer therapy, i.e., Eastern Cooperative Oncology Group (ECOG) performance status ≤ 2. Our hypothesis was that we could find the combination of the most significant biomarkers, which would provide accurate prediction for OS in a real-life setting, and the results may confirm the data of the literature.

## Materials and Methods

### Patients

Blood samples of consecutive patients with locally advanced, recurrent, or metastatic malignant diseases were taken in our clinical chemistry laboratory (Szent Lázár County Hospital, Salgótarján, Hungary) as part of the routine investigation before the initiation of the therapy of the given disease. Obvious symptoms and signs of common infectious diseases were assessed (purulent cough, pulmonary crackles, or symptomatic bacteriuria). Exclusion criteria included suspected infection, hematological malignancy, the lack of at least one biomarker data point, rapid progression (i.e., from laboratory testing, ECOG performance status progressed to 3 before the initiation of anticancer treatment), or death caused by something other than disease progression. Patients with all the following biomarkers available were included in the study: CRP, D-dimer, LDH, albumin, and complete blood count (CBC). Data of 13 excluded patients are given in Supplementary File S1.

### Methods

CRP, LDH, and albumin were measured with commercially available Roche tests on Cobas c501 or Cobas 6000 analyzers (Tokyo, Japan). D-dimer levels were measured by a chemiluminescent immunoassay (PATHFAST, Tokyo, Japan). CBC was determined with Cell-dyn 3700 (Abbott Park, IL, United States and Beckman Unicel DxH600, Miami, FL, United States). The LMR, NLR, and PLR were calculated as the ratio of the lymphocyte count and the monocyte count, the ratio of the neutrophil count and the lymphocyte count, and the ratio of the platelet count and the lymphocyte count, respectively.

### Statistical Analysis

For the purpose of statistical analysis, we used for CRP <5 mg/L (lower level of detection), the value of 4.9 mg/L, and for D-dimer >5 mcg/mL (higher level of detection), the value of 5.1 mcg/mL. All other biomarker values were handled with the measured numeric values. Cut-off determination was performed with the validated “Cutoff Finder” online tool (46). After uploading the tab separated value file (Supplementary File S2), for each biomarker the “Survival Time” was OS or censored OS, the “Survival Event” was the variate of 1 for OS or 0 for censored OS, and the “Method for cut-off determination” was “Survival: significance (log-rank test). Statistical analysis was performed by R Studio Software (47). Semicolon separated value file (Supplementary File-2b.csv) was used.” For each value a comparison was made between the median OS values below and over the cut-off value by the log-rank test. The value with the largest gap and Chi-squared statistics was selected. Comparison of the prognostic groups with Cox proportional hazard regression was performed. Log-rank test was used to detect the differences between survival curves within the prognostic groups in the Kaplan-Meier analysis as well as to assess the significance of the Cox model. Effect size estimation was performed for the Mann-Whitney probe by calculating the so called Eta-squared value. Between 0.06 and 0.14, the effect can be considered medium-sized, while over 0.14 it can be considered large. Power analysis was performed with the “powerCT” function in the “powerSurvEpi” package of the R Studio software. All figures were drawn as vector graphics in Scalable Vector Graphic format in the “ggsurvplot” and “ggforest” functions in the “survminer” package of the R Studio software (47) and edited by Inkscape software (https://inkscape.org). The R-script is available in Supplementary File S3.

OS time was defined as the length of survival from the date of laboratory testing. Survival data measured in months were computed according to Surveillance, Epidemiology, and End Results (SEER) recommendations (https://seer.cancer.gov/survivaltime/SurvivalTimeCalculation.pdf): days between the dates were divided by one twelfth of 365.24. For the median follow-up time calculation, we used a reverse Kaplan-Meier estimator (48).

## Results

### Patient Characteristics

Between July 2016 and August 2019, blood samples of 88 consecutive patients with locally advanced, recurrent, or metastatic malignant disease were analyzed. No common infectious diseases were diagnosed. Data of 13 patients were excluded from the final analysis because of hematological malignancy 1), the lack of any biomarker data (2), death caused by rapid progression before the initiation of anticancer therapy (4), or by other cause of death than disease progression (6). Thus the final retrospective analysis included the data of 75 patients. The shortest censored survival time was 24 months, i.e., the time elapsed since July 2019. As of July 2021, six (8%) patients were still alive. Data of patient characteristics are described in Table 1. Additional data are given in Supplementary Tables S1–S11 in Supplementary File S1.

**TABLE 1:** Characteristics of the 75 patients.

Sex				
	Male	57.3% (43/75)		
	Female	42.6% (32/75)		
Average age				
	Male	62.97 years		
	Female	66.65 years		
Malignancy (n = 75)				TNM stage
	Locally advanced (20/75)			
		HNSCC (8/20)		
			Nasopharynx	cT4cN1cM0
			Hard palate	cT3cN2acM0
			Pharynx	cT2cN0cM0
			Hypopharynx	cT3cNxcM0
				cT3cN0cM0
				cT3cN1cM0
				cT2cN2bcM0
				cT2cN1cM0
		SCLC & hypopharyngeal SCC (1/20)		cT2cN2cM0;cT1cNxcM0
		SCLC (1/20)		cT3cN3cM0
		NSCLC SCC (2/20)		cT4cN2cM0
				cT2cNxcM0
		NSCLC AC (3/20)		cT2cNxcM0
				cT4cN1cM0
				cT3cN2cM0
		GC AC (1/20)		cT3cN1cM0
		PC AC (1/20)		cT4cNxcM0
		CRC (2/20)		
			Transverse colon	cT4cN2cM0
			Rectum	cT4cN1cM0
		OC (1/20)		
			AC	cT3cN1cM0
	Recurrent (6/75)			
		HNSCC (2/6)		
			Tongue	cT2cN1cM0
			Pharynx	cT2cN2acM0
	Recurrent (6/75)			
		GC AC (1/6)		
			Abdominal lymph node	pT3pN2cM0
		BC (3/6)		
			Axillary lymph node	cT1ccN1cM0
			Neck lymph node	pTxcN3cM0
			Local	cT4cNxcM0
	Metastatic (49/75)			
		Parotid SCC (1/49)		
			Suprarenal met.	T3cN2bcM1
		Tongue SCC (1/49)		
			Pulmonary met.	cT1cN2acM1
		Hypopharyngeal SCC (2/49)		
			Pulmonary met.	cT1cN1cM1
			Osseal met.	cT1cN1cM1
		NSCLC AC (4/49)		
			Pulmonary, cerebral met. cT2cN2cM1	cT2cN2cM1
			Pleural carcinosis	cT1ccNxpM1
			Osseal met.	cT3cN2cM1
				cT4cN2cM1
			Pulmonary, osseal met.	pT2pN1pM1
		NSCLC SCC (2/49)		
			Osseal met.	cT4cN2cM1
			Pulmonary, osseal met.	cT3cN1cM1
		GC AC (2/49)		
			Hepatic met.	cT3cN3cM1
			Peritoneal carcinosis	cT3cNxcM1
				cT4cN3cM1
		CRC AC cecal (4/49)		
Malignancy (n = 75)				TNM stage
	Metastatic (49/75)			
			Hepatic met.	pT4pN1pM1
				pT3pN2pM1
			Hep. met., perit. carcinosis	cT4cNxcM1
				cT4cN1pM1
		CRC AC transverse (1/49)		
			Hepatic met.	pT4pN1pM1
		CRC AC sigmoid (1/49)		
			Peritoneal carcinosis	pT3pN2pM1
		CRC AC rectal (8/49)		
			Hepatic met.	cT4cNxpM1
				pT2pN1pM1
				pT2pNxpM1
				cT4cN2pM1
			Hepatic, pulmonary met	pT3pN1pM1
				pT3pN1pM1
				cT4cNxcM1
			Pulmonary met.	cT4cN1cM1
		PC AC (8/49)		
			Pulmonary met.	cTxcN2cM1
			Osseal met.	cTxcNxcM1
			Osseal, cerebral met.	cT2cN2cM1
			Hepatic met.	cT2cNxpM1
				cT2cNxpM1
				cT2cN2pM1
				cT2cN2pM1
				cT2cN1pM1
		Cholecyst AC (1/49)		
			Hepatic met.	pT2pN1pM1
		PCA (3/49)		
			Hep., pulm., osseal met.	pT1ccN1cM1
			Pulmonary, osseal met.	pT2acNxcM1
			Osseal met.	cT2acN1cM1
		Bladder TCC (1/49)		
			Pulmonary met.	pT2bpN2cM1
		BC NST (5/49)		
			Pulmonary, osseal met.	pT4cpN3acM1
				pT1cpN2cM1
			Perit. carcin., osseal met	pT2pN2acM1
			Osseal met.	pT1cpN2acM1
				cT4cN1cM1
		BC neuroendocrine (1/49)		
			Mediastinal, osseal met.	cT4cN1cM1
		OC AC (2/49)		
			Pulmonary met.	cT1bcNxcM1
				cT3cN1cM1

AC, adenocarcinoma; BC, breast cancer; CRC, colorectal cancer; GC, gastric cancer; HNSCC, head and neck squamous cell carcinoma; NSCLC, non-small cell lung cancer; NST, non specified type; OC, ovarian cancer; PC, pancreatic cancer; PCA, prostate adenocarcinoma; SCC, squamous cell carcinoma; SCLC, small cell lung cancer; TCC, transitional cell carcinoma.

### Baseline Biomarkers and Survival

**FIGURE 1:**
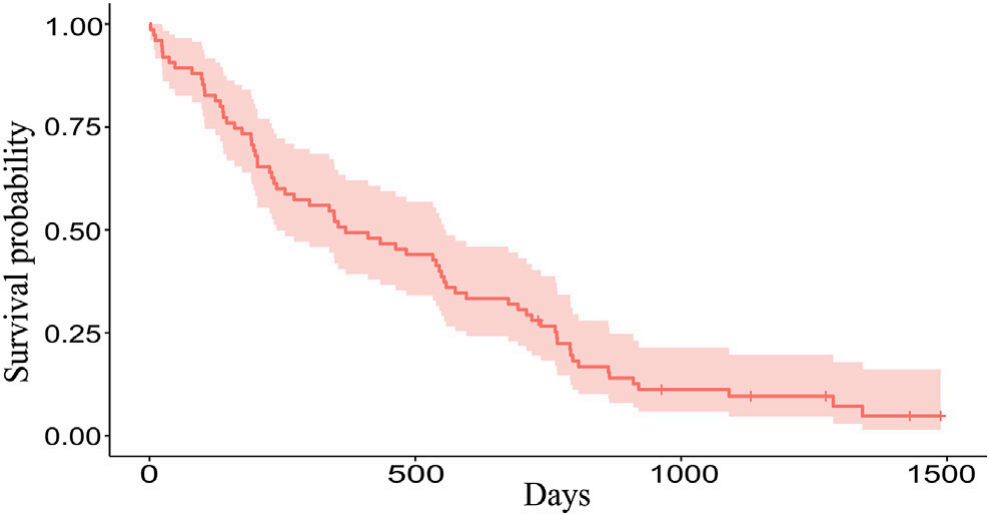
The Kaplan-Meier plot of 75 patients. Overall, 69 patients died and 6 patients are still alive (censored data). Median OS is 369 days (12.12 months), range 2–1488 days (0.06–48.89 months).

The Kaplan-Meier plot was used to determine the median OS and the median follow-up times. With a median follow-up of 46.98 months [95% confidence interval (CI): 37.16–49.28] the median OS was 12.12 months (95% CI: 7.85–18.33) (Figure 1). Mean values of CRP, D-dimer, LDH, albumin, LMR, NLR, and PLR were 28.83 mg/L, 1.70 mcg/mL, 482.12 U/L, 41.62 g/L, 3.41, 4.29, and 168.83, respectively.

#### Determination of Cut-off Values

The following cut-off values were determined for CRP (Chi-squared = 20.85; *p* < 0.001), D-dimer (Chi-squared = 12.94; *p* < 0.001), LDH (Chi-squared = 10.45; *p* < 0.001), albumin (Chi-squared = 15.63; *p* < 0.001), LMR (Chi-squared = 3.45; *p* = 0.063), NLR (Chi-squared = 10.50; *p* < 0.001), and PLR (Chi-squared = 15.17; *p* < 0.001): 30.65 mg/L, 1.98 mcg/mL, 410.50 U/L, 44.35 g/L, 2.65, 4.34, and 168.20, respectively. The three most significant biomarkers were the following: CRP (Eta-squared = 0.188; large power size), albumin (Eta-squared = 0.147; large power size), and PLR (Eta-squared = 0.153; large power size).

#### The Relationship Between the Prognostic Cut-off Values and Survival

**FIGURE 2:**
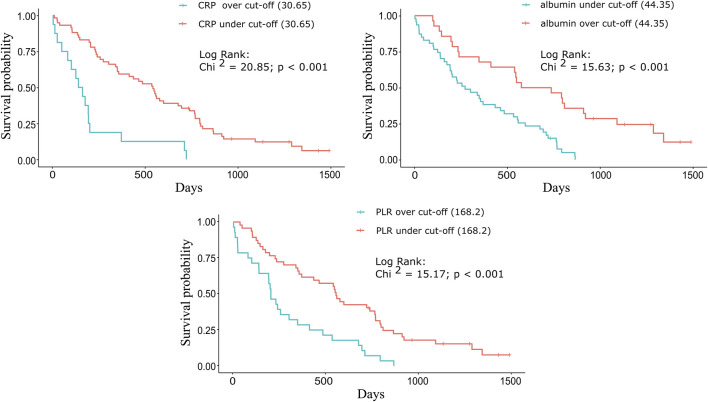
Kaplan-Meier plots for the three significant biomarkers. For **(A)** CRP and **(C)** PLR, longer survivals were found below the cut-off (30.65 mg/L and 168.20) values: 539 vs. 149 days (17.71 vs. 4.89 months) and 554 vs. 203 days (18.20 vs. 6.67 months). For **(B)** albumin, longer survival was found above the cut-off (44.35 g/L) value: 655.5 vs. 272 days (21.54 vs. 8.94 months).

For each biomarker, a Kaplan-Meier plot was used to compare the median OS of the groups above and below the cut-off value (Figures 2A–C). For CRP and PLR (Figures 2A,C), longer survivals were found below than above the cut-off value. For albumin (Figure 2B), longer survival was found above the cut-off values (Table 2).

**TABLE 2:** Comparison of the median OS based on the cut-off value for each significant biomarker.

	CRP (mg/L)		Albumin (g/L)		PLR	
Cut-off value	>30.65	≤30.65	≤44.35	>44.35	>168.20	≤168.20
*n* =	16	59	47	28	28	47
Median OS (months)	4.89	17.71	8.94	21.54	6.67	18.20
Mann-Whitney test (Z statistic)	3.75		3.32		3.38	
*p*-value	<0.001		<0.001		<0.001	

### Classification of Patients Into Risk Groups

**FIGURE 3:**
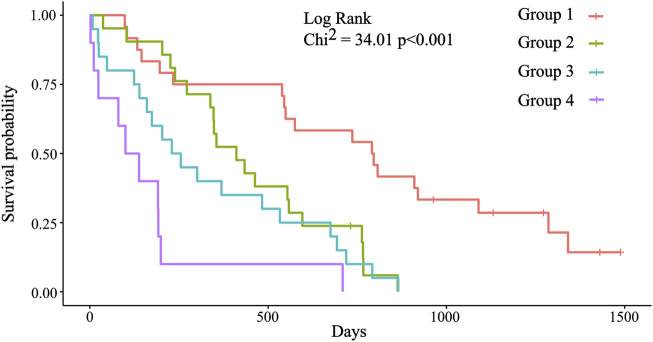
Kaplan-Meier survival plots for the four prognostic groups. Group 1: Median OS = 793.5 days (26.07 months); group 2: Median OS = 411.0 days (13.50 months); group 3: Median OS = 242.5 days (7.97 months); group 4: Median OS = 119 days (3.91 months). Significant differences were detected between group 1 (reference) and groups 2, 3, and 4 (*p* = 0.003; *p* < 0.001; *p* < 0.001).

With the combination of three biomarkers, prognostic groups were created independently from stage, histology, and time to progression on first line therapy (Supplementary File S1). Four prognostic groups were formed based on the cut-off values of each biomarker. Group 1: No biomarker with out-of-range value (ORV), defined by the cut-off value; group 2: One ORV biomarker; group 3: Two ORV biomarkers; and group 4: Three ORV biomarkers (Table 3). Significant differences were detected between these groups (Table 4, Figure 3). The likelihood ratio test of the Cox model regression parameters for the four groups was 29.5 (*p* < 0.001).

**TABLE 3:** The four prognostic groups based on the established cut-off values of the selected three biomarkers.

	Group 1	Group 2			Group 3			Group 4
CRP (mg/L)	≤30.65	**>30.65**	≤30.65	≤30.65	**>30.65**	**>30.65**	≤30.65	**>30.65**
Albumin (g/L)	>44.35	>44.35	**≤44.35**	>44.35	**≤44.35**	>44.35	**≤44.35**	**≤44.35**
PLR	≤168.20	≤168.20	≤168.20	**> 168.20**	≤168.20	**> 168.20**	**> 168.20**	**> 168.20**

Out-of-range values (ORV) of the biomarkers are in bold.

**TABLE 4:** Prognostic significance of the four prognostic groups.

Group	n =	Median OS (m)	HR (95%CI)	*p*-value	Power (95%CI)
1	24	26.07	1	-	-
2	21	13.50	3.0 (1.5–6.2)	0.003	0.896 (0.242–0.997)
3	20	7.97	4.1 (2.0–8.3)	<0.001	0.976 (0.570–0.999)
4	10	3.91	10.2 (4.2–24.6)	<0.001	0.999 (0.981–1)

### Evaluation of the Survival Prediction of Three Biomarkers

We compared the median OS of groups with one ORV biomarker with that of groups with two and three ORV biomarkers using the Mann-Whitney test and Z statistic (Table 5). The comparison of the group of ORV albumin with the group of ORV CRP and albumin values indicated a significant difference (*p* = 0.04; Eta-squared = 0.067; medium power size). A similar significance was detected comparing the group ORV albumin with the group of ORV CRP and PLR (*p* = 0.026; Eta-squared = 0.087; medium power size). The cases in the groups of ORV CRP and PLR also had ORV albumin.

**TABLE 5:** Survival prediction of the usage of two ORV biomarkers* compared to the usage of one ORV biomarker.

	CRP > 30.65 mg/L with albumin ≤ 44.35 g/L			CRP > 30.65 mg/L with PLR > 168.20#			Albumin ≤ 44.35 g/L with PLR > 168.20		
n =	16			10			24		
Median OS (m)	4.89			3.91			6.42		
Ref. §	CRP > 30.65	Alb. ≤ 44.35	PLR > 168.20	CRP > 30.65	Alb. ≤ 44.35	PLR > 168.20	CRP > 30.65	Alb. ≤ 44.35	PLR > 168.20
M-W test Z statistic	0	2.05	1.32	0.47	2.22	1.66	−0.91	1.14	0.26
*p*-value	1	**0.040**	0.188	0.635	**0.026**	0.097	0.362	0.253	0.790

*Irrespective of the third biomarker value.

#Group with elevated ORV CRP and PLR values also had decreased ORV albumin values. Consequently no patient with ORV CRP and PLR with normal albumin was present.

§Each reference group has one ORV biomarker.

No significant differences were found between the groups with two ORV biomarkers with three ORV biomarkers.

## Discussion

In this retrospective and confirmatory analysis, we applied seven routinely measured clinical laboratory parameters (CRP, albumin, D-dimer, LDH, and based on CBC, calculated LMR, NLR and PLR) to a consecutive real-life patient population of locally advanced, recurrent, and metastatic malignant diseases at a single institution (Szent Lázár County Hospital), and searched for the most significant combination. These parameters and some of their combinations have already been proven to be independent prognostic factors for cancer.

Chronic low grade and intensity inflammation might precede malignant transformation and is considered to be a predisposing factor in cancer development (49). CRP is regarded as a biomarker of acute and chronic inflammation. Without other inflammatory processes, CRP may be increased (upper limit of normal CRP < 5 mg/L) in malignant diseases. In early-stage malignant diseases, a baseline normal CRP level correlates with longer OS. In locally advanced and metastatic settings, lower baseline CRP correlates with better prognosis (16).

Formation of serum albumin is determined by the osmotic colloid pressure, by the inflammatory and nutritional state of the body, and by hormonal factors. In cases of patients with localized malignant diseases both moderate hypoalbuminemia (<34 g/L) and a normal albumin level can occur. However, during disease progression, weight loss is accompanied by a significant decrease of albumin level. In a locally advanced and/or metastatic setting, serum albumin level diminishes independently in the presence of malnutrition. Lower baseline albumin suggests poor survival (19).

Elevated PLR (e.g., ≥200; >146.2; ≥180; >150; >220; >181.24) was proven to be an adverse prognostic factor in various cancers (34–45).

Here, the three most significant biomarkers were found: CRP, albumin, and PLR (Table 2), and stratification of the patients into one of the four groups was performed according to the number of ORV biomarkers (Table 3). We found that these prognostic groups enable the identification of good, moderate, intermediate, and poor OS patients with reasonable accuracy (Figure 3, Table 4). Based on our results, we can confirm that a combination of biomarkers probably has a better prognostic value than any of the single biomarkers (Table 5). Other prognostic threshold values published in previous studies were comparable to our results (16, 19, 34–45).

Our analysis has some limitations. First, the patient population for this small-scale retrospective analysis is histologically heterogenous. Second, regarding the stage, these unbalanced cohorts of locally advanced, recurrent, or metastatic diseases are also heterogenous. Third, the identified cut-off values by this study for CRP, albumin, and PLR are slightly different from those used by other studies, therefore they need to be validated in a large-scale prospective study. Fourth, there are multiple factors that could have a possible influence on the OS of patients that were not monitored in our analysis.

## Conclusion

Based on our analysis, we can confirm that the combination of serum biomarkers measured at baseline would provide accurate estimation for OS in real-life advanced cancer patients. We were able to establish consistent prognostic groups using the most significant three biomarkers. The OS was significantly different in each of the prognostic groups developed. One advantage of our study is that these parameters can be routinely measured without additional costs. We are persuaded that the prognostic significance of these and other biomarker patterns, and their role in relation to the well-established prognostic systems, warrants further investigation and validation in large prospective cohorts of real-life cancer patients.

## Data Availability

The datasets presented in this study can be found in online repositories. The names of the repository/repositories and accession number(s) can be found in the article/Supplementary Material.

## Supplementary Material

Supplementary File S1 Summary of the data of 13 excluded patients. Additional data of 75 patients demonstrated in Supplementary Tables S1–S11.

Supplementary File S2 The biomarker values in a tab separated value file for “Cutoff Finder.”

Supplementary File S3 The biomarker and survival values in semicolon separated value file for the analysis inR Studio Software.

Supplementary File S4 R‐code is extended with the script of the KM curves for the prognostic groups.
