# Corrigendum: Overall Survival Prediction of Advanced Cancer Patients by Selection of the Most Significant Baseline Serum Biomarker Combination

**DOI:** 10.3389/pore.2022.1610517

**Published:** 2022-05-18

**Authors:** Daniel Deme, Sandor Kovacs, Andras Telekes

**Affiliations:** ^1^ Department of Medical Oncology, Szent Lázár County Hospital, Salgótarján, Hungary; ^2^ Department of Economical and Financial Mathematics, University of Debrecen, Debrecen, Hungary

**Keywords:** overall survival, advanced cancer, serum biomarkers, prognostic importance, CRP, albumin, PLR

In the original article, there was a mistake in Table 2. In the last column of PLR the wrong patient number is 57. The correct patient number is 47. The corrected Table 2 appears below.

**TABLE 2:** Comparison of the median OS based on the cut-off value for each significant biomarker.

	CRP (mg/L)		Albumin (g/L)		PLR	
Cut-off value	>30.65	≤30.65	≤44.35	>44.35	>168.20	≤168.20
*n*=	16	59	47	28	28	47
Median OS (months)	4.89	17.71	8.94	21.54	6.67	18.20
Mann-Whitney test (Z statistic)	3.75		3.32		3.38	
*p*-value	<0.001		<0.001		<0.001	

The authors apologize for this error and state that this does not change the scientific conclusions of the article in any way. The original article has been updated.

