# Supplementary material for: Overall Survival Prediction of Advanced Cancer Patients by Selection of the Most Significant Baseline Serum Biomarker Combination
Source: Pathol Oncol Res. 2022 Jan 31;28:1610004. doi: 10.3389/pore.2022.1610004 (PMC8842665; doi:10.3389/pore.2022.1610004)
Supplement: Supplementary file 1 [file DataSheet4.pdf]

```
# load packages
#makes sure the following packages are installed in your R Studio
library(MASS)
library(nlme)
library(splines)
library(survival)
library(survminer)
library(ranger)
library(ggplot2)
library(ggfortify)
library(dplyr)
library(plotROC)
library(powerSurvEpi)
library(coin)

# load data, Don't forget to change the directory of the source file!!!
adat <- read.csv2("~/pen/kutattan/munka/deme/ujra3/adatok.csv")

#logarithmize data with base 10
 for (i in 1:8) adat[,i]<-(as.numeric(adat[,i]))


#create groups with respect to cut-offs
adat2<-adat
adat2$crp = ifelse(as.numeric(adat2$crp)<=30.65, 0, 1)
adat2$alb = ifelse(adat2$alb<=44.35, 1, 0)
adat2$nlr = ifelse(adat2$nlr<=4.341, 0, 1)
adat2$ddim = ifelse(adat2$ddim<=1.985, 0, 1)
adat2$lmr = ifelse(adat2$lmr<=2.649, 1, 0)
adat2$plr = ifelse(adat2$plr<=168.2, 0, 1)
adat2$ldh = ifelse(adat2$ldh<=410.5, 0, 1)

# KM curve and median survival for increased number of biomarkers
survobject<-Surv(time=adat2$surv,event=adat2$cenz1)
fit1 <- survfit(survobject ~ crp, data = adat2)
ggsurvplot(fit1, data = adat2, pval = TRUE)
sdc<-survdiff(survobject ~ crp, data = adat2)
sdc$chisq

# KM curve and median survival for increased number of biomarkers
survobject<-Surv(time=adat2$surv,event=adat2$cenz1)
fit1 <- survfit(survobject ~ alb, data = adat2)
ggsurvplot(fit1, data = adat2, pval = TRUE)
sdc<-survdiff(survobject ~ alb, data = adat2)
sdc$chisq

# KM curve and median survival for increased number of biomarkers
survobject<-Surv(time=adat2$surv,event=adat2$cenz1)
fit1 <- survfit(survobject ~ lmr, data = adat2)
ggsurvplot(fit1, data = adat2, pval = TRUE)
sdc<-survdiff(survobject ~ lmr, data = adat2)
sdc$chisq

# KM curve and median survival for increased number of biomarkers
survobject<-Surv(time=adat2$surv,event=adat2$cenz1)
fit1 <- survfit(survobject ~ nlr, data = adat2)
ggsurvplot(fit1, data = adat2, pval = TRUE)
sdc<-survdiff(survobject ~ nlr, data = adat2)
sdc$chisq

# KM curve and median survival for increased number of biomarkers
```

```
survobject<-Surv(time=adat2$surv,event=adat2$cenz1)
fit1 <- survfit(survobject ~ plr, data = adat2)
ggsurvplot(fit1, data = adat2, pval = TRUE)
sdc<-survdiff(survobject ~ plr, data = adat2)
sdc$chisq

# KM curve and median survival for increased number of biomarkers
survobject<-Surv(time=adat2$surv,event=adat2$cenz1)
fit1 <- survfit(survobject ~ ddim, data = adat2)
ggsurvplot(fit1, data = adat2, pval = TRUE)
sdc<-survdiff(survobject ~ ddim, data = adat2)
sdc$chisq

# KM curve and median survival for increased number of biomarkers
survobject<-Surv(time=adat2$surv,event=adat2$cenz1)
fit1 <- survfit(survobject ~ ldh, data = adat2)
ggsurvplot(fit1, data = adat2, pval = TRUE)
sdc<-survdiff(survobject ~ ldh, data = adat2)
sdc$chisq

surv_pvalue(fit1)

#create biomarker groups based only on the 3 most significant result
adat2$sum<-adat2$crp+adat2$alb+adat2$plr
adat2$sum<-as.factor(adat2$sum)

#median OS for groups
group1<-adat2[adat2$sum==0,]
group2<-adat2[adat2$sum==1,]
group3<-adat2[adat2$sum==2,]
group4<-adat2[adat2$sum==3,]

# KM curve and median survival for biomarker groups
survobject<-Surv(time=adat2$surv,event=adat2$cenz1)
fit1 <- survfit(survobject ~ sum, data = adat2)
ggsurvplot(fit1, data = adat2, pval = TRUE)
sdc<-survdiff(survobject ~ sum, data = adat2)
sdc$chisq


mos<-
c(median(group1$surv),median(group2$surv),median(group3$surv),median(group4
$surv))
mos

#fit a Cox model with the "sum" showing the number of increased biomarkers
coxFit <- coxph(Surv(surv, cenz1) ~ sum, data = adat2, x = TRUE)
survobject<-Surv(time=adat2$surv,event=adat2$cenz1)

# survival curve plotting and Hazard Ratio calculation
plot(survfit(coxFit))
ggforest(coxFit, data = adat2)

#Power analyses for Cox-regression

powerCT.default(nE = 21,
                nC = 24,
                pE = 0.95,
                pC = 0.79,
                RR = 3,
                alpha = 0.05)
```

```
#lowerCI
powerCT.default(nE = 21,
                nC = 24,
                pE = 0.95,
                pC = 0.79,
                RR = 1.5,
                alpha = 0.05)

#upperCI
powerCT.default(nE = 21,
                nC = 24,
                pE = 0.95,
                pC = 0.79,
                RR = 6.2,
                alpha = 0.05)



powerCT.default(nE = 20,
                nC = 24,
                pE = 1.00,
                pC = 0.79,
                RR = 4,
                alpha = 0.05)

#lowerCI
powerCT.default(nE = 20,
                nC = 24,
                pE = 1.00,
                pC = 0.79,
                RR = 2,
                alpha = 0.05)

#upperCI
powerCT.default(nE = 20,
                nC = 24,
                pE = 1.00,
                pC = 0.79,
                RR = 8.3,
                alpha = 0.05)



powerCT.default(nE = 10,
                nC = 24,
                pE = 1.00,
                pC = 0.79,
                RR = 10,
                alpha = 0.05)

#lowerCI
powerCT.default(nE = 10,
                nC = 24,
                pE = 1.00,
                pC = 0.79,
                RR = 4.2,
                alpha = 0.05)
```

```
#upperCI
powerCT.default(nE = 20,
                nC = 24,
                pE = 1.00,
                pC = 0.79,
                RR = 24.6,
                alpha = 0.05)


#1. crp & alb vs. only crp

class1<-adat2[adat2$crp==1,]
class2<-adat2[adat2$crp==1&adat2$alb==1,]
class1$grp=1
class2$grp=2
one<-rbind(class1,class2)

#median OS for the separate groups
median(class1$surv)
median(class2$surv)

#Group sizes
nrow(class1)
nrow(class2)

#MW-test
mw<-wilcox_test(one$surv ~ as.factor(one$grp))



#2. crp & alb vs. only alb

class1<-adat2[adat2$alb==1,]
class2<-adat2[adat2$crp==1&adat2$alb==1,]
class1$grp=1
class2$grp=2
one<-rbind(class1,class2)

#median OS for the separate groups
median(class1$surv)
median(class2$surv)

#Group sizes
nrow(class1)
nrow(class2)

#MW-test
mw<-wilcox_test(one$surv ~ as.factor(one$grp))


#3. crp & plr vs. only plr

class1<-adat2[adat2$plr==1,]
class2<-adat2[adat2$crp==1&adat2$plr==1,]
class1$grp=1
class2$grp=2
one<-rbind(class1,class2)

#median OS for the separate groups
median(class1$surv)
median(class2$surv)
```

```
#Group sizes
nrow(class1)
nrow(class2)

#MW-test
mw<-wilcox_test(one$surv ~ as.factor(one$grp))
mw


#4. crp & plr vs. only crp

class1<-adat2[adat2$crp==1,]
class2<-adat2[adat2$crp==1&adat2$plr==1,]
class1$grp=1
class2$grp=2
one<-rbind(class1,class2)

#median OS for the separate groups
median(class1$surv)
median(class2$surv)

#Group sizes
nrow(class1)
nrow(class2)

#MW-test
mw<-wilcox_test(one$surv ~ as.factor(one$grp))
mw


#5. crp & plr vs. only alb

class1<-adat2[adat2$alb==1,]
class2<-adat2[adat2$crp==1&adat2$plr==1,]
class1$grp=1
class2$grp=2
one<-rbind(class1,class2)

#median OS for the separate groups
median(class1$surv)
median(class2$surv)

#Group sizes
nrow(class1)
nrow(class2)

#MW-test
mw<-wilcox_test(one$surv ~ as.factor(one$grp))
mw

#6. crp & alb vs. only plr

class1<-adat2[adat2$plr==1,]
class2<-adat2[adat2$crp==1&adat2$alb==1,]
class1$grp=1
class2$grp=2
one<-rbind(class1,class2)

#median OS for the separate groups
median(class1$surv)
```

```
median(class2$surv)

#Group sizes
nrow(class1)
nrow(class2)

#MW-test
mw<-wilcox_test(one$surv ~ as.factor(one$grp))
mw


#7. crp & alb vs. crp,Alb,plr

class1<-adat2[adat2$crp==1&adat2$alb==1&adat2$plr==1,]
class2<-adat2[adat2$crp==1&adat2$alb==1,]
class1$grp=1
class2$grp=2
one<-rbind(class1,class2)

#median OS for the separate groups
median(class1$surv)
median(class2$surv)

#Group sizes
nrow(class1)
nrow(class2)

#MW-test
mw<-wilcox_test(one$surv ~ as.factor(one$grp))
mw


#8. crp & plr vs. crp,Alb,plr

class1<-adat2[adat2$crp==1&adat2$alb==1&adat2$plr==1,]
class2<-adat2[adat2$crp==1&adat2$plr==1,]
class1$grp=1
class2$grp=2
one<-rbind(class1,class2)

#median OS for the separate groups
median(class1$surv)
median(class2$surv)

#Group sizes
nrow(class1)
nrow(class2)

#MW-test
mw<-wilcox_test(one$surv ~ as.factor(one$grp))
mw

#9. Alb & plr vs. crp,Alb,plr

class1<-adat2[adat2$crp==1&adat2$alb==1&adat2$plr==1,]
class2<-adat2[adat2$alb==1&adat2$plr==1,]
class1$grp=1
class2$grp=2
one<-rbind(class1,class2)

#median OS for the separate groups
```

```
median(class1$surv)
median(class2$surv)

#Group sizes
nrow(class1)
nrow(class2)

#MW-test
mw<-wilcox_test(one$surv ~ as.factor(one$grp))
mw
```

10. Alb & plr vs. crp

```
class1<-adat2[adat2$crp==1,]
class2<-adat2[adat2$alb==1&adat2$plr==1,]
class1$grp=1
class2$grp=2
one<-rbind(class1,class2)

#median OS for the separate groups
median(class1$surv)
median(class2$surv)

#Group sizes
nrow(class1)
nrow(class2)

#MW-test
mw<-wilcox_test(one$surv ~ as.factor(one$grp))
mw
```

11. Alb & plr vs. alb

```
class1<-adat2[adat2$alb==1,]
class2<-adat2[adat2$alb==1&adat2$plr==1,]
class1$grp=1
class2$grp=2
one<-rbind(class1,class2)

#median OS for the separate groups
median(class1$surv)
median(class2$surv)

#Group sizes
nrow(class1)
nrow(class2)

#MW-test
mw<-wilcox_test(one$surv ~ as.factor(one$grp))
mw
```

12. Alb & plr vs. plr

```
class1<-adat2[adat2$plr==1,]
class2<-adat2[adat2$alb==1&adat2$plr==1,]
class1$grp=1
class2$grp=2
one<-rbind(class1,class2)
```

```
#median OS for the separate groups
median(class1$surv)
median(class2$surv)

#Group sizes
nrow(class1)
nrow(class2)

#MW-test
mw<-wilcox_test(one$surv ~ as.factor(one$grp))
mw
```

13. Alb & plr & CRP vs. CRP

```
class1<-adat2[adat2$crp==1,]
class2<-adat2[adat2$alb==1&adat2$plr==1&adat2$crp==1,]
class1$grp=1
class2$grp=2
one<-rbind(class1,class2)

#median OS for the separate groups
median(class1$surv)
median(class2$surv)

#Group sizes
nrow(class1)
nrow(class2)

#MW-test
mw<-wilcox_test(one$surv ~ as.factor(one$grp))
mw
```

14. Alb & plr & CRP vs. alb

```
class1<-adat2[adat2$alb==1,]
class2<-adat2[adat2$alb==1&adat2$plr==1&adat2$crp==1,]
class1$grp=1
class2$grp=2
one<-rbind(class1,class2)

#median OS for the separate groups
median(class1$surv)
median(class2$surv)

#Group sizes
nrow(class1)
nrow(class2)

#MW-test
mw<-wilcox_test(one$surv ~ as.factor(one$grp))
mw
```

15. Alb & plr & CRP vs. plr

```
class1<-adat2[adat2$plr==1,]
class2<-adat2[adat2$alb==1&adat2$plr==1&adat2$crp==1,]
class1$grp=1
class2$grp=2
one<-rbind(class1,class2)
```

```
#median OS for the separate groups
median(class1$surv)
median(class2$surv)

#Group sizes
nrow(class1)
nrow(class2)

#MW-test
mw<-wilcox_test(one$surv ~ as.factor(one$grp))
mw



#Mann-whitney probe on the groups

#CRP

class1<-adat2[adat2$crp==0,]
class2<-adat2[adat2$crp==1,]
class1$grp=1
class2$grp=2
one<-rbind(class1,class2)

#median OS for the separate groups
median(class1$surv)
median(class2$surv)

#Group sizes
nrow(class1)
nrow(class2)

#MW-test
mw<-wilcox_test(one$surv ~ as.factor(one$grp))
mw

#Alb

class1<-adat2[adat2$alb==0,]
class2<-adat2[adat2$alb==1,]
class1$grp=1
class2$grp=2
one<-rbind(class1,class2)

#median OS for the separate groups
median(class1$surv)
median(class2$surv)

#Group sizes
nrow(class1)
nrow(class2)

#MW-test
mw<-wilcox_test(one$surv ~ as.factor(one$grp))
mw


#Plr

class1<-adat2[adat2$plr==0,]
```

```
class2<-adat2[adat2$plr==1,]
class1$grp=1
class2$grp=2
one<-rbind(class1,class2)

#median OS for the separate groups
median(class1$surv)
median(class2$surv)

#Group sizes
nrow(class1)
nrow(class2)

#MW-test
mw<-wilcox_test(one$surv ~ as.factor(one$grp))
mw
```
