# Supplementary material for: Overall Survival Prediction of Advanced Cancer Patients by Selection of the Most Significant Baseline Serum Biomarker Combination
Source: Pathol Oncol Res. 2022 Jan 31;28:1610004. doi: 10.3389/pore.2022.1610004 (PMC8842665; doi:10.3389/pore.2022.1610004)
Supplement: Supplementary file 3 [file DataSheet1.pdf]

SUPPLEMENTARY FILE - 1

Abbreviations: ACC = adenocarcinoma; AF = atrial fibrillation; ALC = absolute lymphocyte count; AMC = absolute monocyte count; ANC = absolute neutrophyl count; BC = breast cancer; BMI = body mass index; COVID19 = Coronavirus Disease 2019; CF = cardiac failure; CRC = colorectal cancer; CRF = chronic renal failure; CRP = C-reactive protein; D-dimer = cross-linked fibrin degradation products; ECOG = Eastern Cooperative Oncology Group; ED = extensive disease (SCLC); GC = gastric cancer; HNSCC = head and neck squamous cell carcinoma; hypoph = hypopharyngeal cancer; IHD = ischemic heart disease; LA = locally advanced; LDH = lactate dehydrogenase; LMR = lymphocyte to monocyte ratio; mBC = metastatic breast cancer; mCRC = metastatic colorectal cancer; mCRPC = metastatic castration resistant prostate cancer; Met = metastatic; mGC = metastatic gastric cancer; mHSPC = metastatic hormon sensitive prostate cancer; mOC = metastatic ovarian cancer; nasoph = nasopharyngeal cancer; NLR = neutrophyl to lymphocyte ratio; NSCLC = non small cell lung cancer; NST = non specified type; OC = ovarian cancer; OS = overall survival; PC = pancreatic cancer; PLR = platelet to lymphocyte ratio; Pts. No. = patients number (in the original cohort from 1-88); Rec = recurrent; SCC = squamous cell carcinoma; SCLC = small cell lung cancer; TCC = transitional cell carcinoma

Summary of the data of 13 patients were excluded from the final analysis

A patient with metastatic uterine sarcoma developed a pathological fracture, and during this period her disease has progressed to death before the initiation of chemotherapy. Other patient with metastatic colorectal cancer had chronic lymphoid leukaemia, therefore the analyzis of LMR, NLR and PLR were not applicable. A metastatic rectal cancer patient had failure of albumin measurement and a metastatic prostate cancer patient had failure of D-dimer measurement before the first line chemotherapy. A patient with metastatic non small cell lung cancer (NSCLC) has progressed rapidly to poor Eastern Cooperative Oncology Group (ECOG) 3 performance status and an other patient with metastatic pancreatic cancer had demonstrated poor ECOG 3 performance status on the contrary of nutritional therapy before the first line chemotherapy. A patient of metastatic colorectal cancer died due to a car accident. A patient with locally advanced nasopharyngeal carcinoma died due to acute renal failure. A patient with metastatic prostate cancer progressed rapidly, thus he was unable to receive chemotherapy. Three patients died due to febrile neutropenia. One patient with metastatic breast cancer died due to severe COVID19 pneumonia.

List of Supplementary Tables 1-11.:

Supplementary Table 1. Description of the patient cohort (n=75)
Supplementary Table 2. Summary of the time interval between blood sampling and the therapy started
Supplementary Table 3. Description of biomarker values and overall survival data
Supplementary Table 4. Description of the therapy administered
Supplementary Table 5. Description of comorbidities
Supplementary Table 6. Data of the six cenzored patients
Supplementary Table 7. Distribution of the patients after stratification into four prognostic groups
Supplementary Table 8. Summary of the patient characterstics of the Group 1 (n=24)
Supplementary Table 9. Summary of the patient characterstics of the Group 2 (n=21)
Supplementary Table 10. Summary of the patient characterstics of the Group 3 (n=20)
Supplementary Table 11. Summary of the patient characterstics of the Group 4 (n=10)

Supplementary Table 1. Description of the patient cohort (n=75)

Pts. No.	Sex	Age (ys)	Neoplasia	Recurr/Met.	ECOG	BMI
1	M	74	LA-NSCLC ACC	-	1	25.95
2	M	37	mPC ACC	pulmonary	1	17.14
4	M	60	mHSPC ACC	bone	1	21.88
5	M	62	mNSCLC ACC	pulmonary, brain	0	27.61
6	M	64	mGC ACC	liver	2	31.41
7	M	71	mGC ACC	peritoneal cc.	2	28.34
9	F	59	Recurrent BC NST	axillary recurrence	0	24.03
10	M	53	mPC ACC	pulmonary, brain	1	19.03
11	M	67	mCRC (coecal) ACC	liver	0	32.96
12	M	78	mCRPC ACC	bone, liver, pulmonary	1	25.86
14	M	67	mBC NST	non-regional lymphnode met. (neck)	1	38.21
15	M	82	mCRPC ACC	bone, pulmonary	0	26.75
16	F	58	LA-HNSCC (tongue)	-	1	14.82
17	F	77	mNSCLC ACC	pleural carcinosis	0	34.26
18	M	59	mCRC (rectal) ACC	liver	1	22.69
19	M	52	Recurrent HNSCC	laryngeal recurrence	0	19.79
20	M	62	LA-CRC (rectal) ACC	-	1	23.26
21	F	46	mNSCLC SCC	bone	1	19.33
22	M	60	Recurrent GC ACC	retroperitoneal lymphnodes	0	21.79
23	F	62	mBC NST	bone, pulmonary	1	17.93
24	M	57	LA-HNSCC (hypoph.)	-	1	19.61
25	F	68	mNSCLC ACC	bone	0	24.22
26	F	82	LA-HNSCC (hard palate)	-	2	15.82
27	M	54	mCRC (coecal) ACC	peritoneal carcinosis, liver	0	30.81
28	M	59	mNSCLC ACC	bone	2	22.31
29	F	72	mOC ACC	pulmonary	2	29.78
30	M	66	mCRC (sigmoid) ACC	peritoneal carcinosis	0	25.93
31	M	61	mHNSCC (tongue)	pulmonary	2	18.83
32	F	76	mOC ACC	pulmonary	2	31.99
33	M	81	LA-HNSCC (larynx)	-	1	19.47
35	M	49	LA-HNSCC (hypoph)	-	2	14.03
36	M	54	mHNSCC (parotid)	adrenal	0	26.47
37	F	60	mPC ACC	liver	0	18.07
38	M	41	mCRC (transverse) ACC	liver	1	27.43
39	F	56	mBC NST	bone, pulmonary	0	24.39
40	M	67	LA-NSCLC SCC	-	2	25.48
41	M	66	mCRC (rectal) ACC	liver	1	36.7
42	M	66	mCRC (rectal) ACC	pulmonary, liver	1	19.14
45	F	66	mCRC (rectal) ACC	pulmonary, liver	1	25.89
46	F	68	mCRC (rectal) ACC	liver	0	23.83
47	M	82	mCRPC ACC	bone	0	38.06
48	M	62	mBladder TCC	pulmonary	1	20.83
49	M	60	mGC ACC	peritoneal carcinosis, bone	2	17.47
50	M	59	mPC ACC	liver	1	28.68

Supplementary Table 1. (continued)

Pts. No.	Sex	Age (ys)	Neoplasia	Recurr/Met.	ECOG	BMI
51	M	51	mHNSCC (hypoph)	pulmonary	0	28.06
52	M	62	mPC ACC	liver	1	20.4
53	F	67	LA-NSCLC ACC	-	1	26.84
54	M	73	mCRC (coecal) ACC	liver	1	19.6
55	F	60	mBC NST	peritoneal carcinosis	2	27.92
56	F	74	LA-OC ACC	-	2	23.34
57	M	46	LA-HNSCC (hypoph)	-	0	21.05
58	M	59	LA-HNSCC (hypoph)	-	1	25.16
59	F	59	mBC NST	bone	1	26.09
60	F	55	LA-NSCLC ACC	-	0	49.13
61	M	63	LA-HNSCC (hypoph)	-	1	22.13
62	F	62	mCRC (rectal) ACC	pulmonary	1	19.82
64	F	76	LA-GC ACC	-	2	28.34
65	M	79	mCRC (coecal) ACC	peritoneal carcinosis, liver	1	21.3
67	M	81	mCRC (rectal) ACC	pulmonary, liver	2	28.39
68	F	54	mNSCLC ACC	pulmonary, bone	1	30.08
69	M	68	mCRC (rectal) ACC	liver	1	20.83
70	F	69	LA-CRC (transverse) ACC	-	2	28.55
72	F	65	mPC ACC	liver	1	54.52
73	F	60	mNSCLC SCC	pulmonary, bone	1	28.83
75	M	70	ED-SCLC	-	1	35.26
76	F	70	Recurrent BC NST	local recurrence	1	24.61
77	F	62	LA-HNSCC (nasoph)	-	2	17.12
78	M	49	LA-PC ACC	-	1	23.62
79	F	74	mPC ACC	liver	2	26.22
80	F	65	LA-NSCLC ACC	-	1	23.63
81	F	65	mBC neuroendocrine	mediastinal, bone	1	19.3
84	M	59	mHNSCC (hypoph)	bone	1	19.29
85	F	73	mBC NST	bone	1	23.88
87	F	78	mCholecyst ACC	liver	1	26.84
88	F	76	ED SCLC & HNSCC(hypoph)		2	19.03

Supplementary Table 2. Summary of the time interval between blood sampling and the therapy started

Pts. No.	n =	Interval	Notes
64;*	1	-	No consent to therapy
1; 26;	2	4 months	Patients postponed the planned radiotherapy
25;	1	63 days	Patient hesitated to start the therapy
47;	1	23 days	
4; 23; 62;	3	22 days	
50;	1	13 days	
81;	1	12 days	
56;	1	7 days	
79;	1	6 days	
59; 72;	2	5 days	
87; 88;	2	3 days	
32; 36; 45;	3	2 days	
7; 11; 46; 73;	4	1 day	
2; 5; 6; 9; 10; 12; 14; 15; 16; 17; 18; 19; 20; 21; 22; 24; 27; 28; 29; 30; 31; 33; 35; 37; 38; 39; 40; 41; 42; 48; 49; 51; 52; 53; 54; 55; 57; 58; 60; 61; 65; 67; 68; 69; 70; 75; 76; 77; 78; 80; 84; 85;	52	0 days	Blood sampling was on the same day of therapy

* = for this patient surgery, radiotherapy or palliative chemotherapy were offered, but consent to either therapy was not given

Supplementary Table 3. Description of biomarker values and overall survival data

**Pts. No.**	**CRP (<5mg/L)**	**D-dimer (<0.5mcg/mL)**	**LDH 240-480 U/L**	**Albumin (<34g/L)**	ALC (1.5-3.5G/L)	ANC (2-7.5G/L)	AMC (0.2-0.8G/L)	PLT (150-400G/L)	**LMR**	**NLR**	**PLR**	**OS (days)**	**OS (m)**	**Censored (Y/N)**
1	**56.6**	**1.98**	362	38.5	**1.43**	4.02	0.6	206	2.38	2.81	144.06	719	23.62	N
2	**78.6**	**1.17**	414	40.9	1.8	**10.8**	**1.14**	262	1.58	6.00	145.56	124	4.07	N
4	<5	**1.66**	324	43.7	1.59	3.45	**0.91**	225	1.75	2.17	141.51	348	11.43	N
5	**19.9**	**1.36**	324	39.7	3.46	6.12	0.77	331	4.49	1.77	95.66	763	25.07	N
6	**117.7**	**>5**	**575**	36.2	1.5	**9.89**	0.77	187	1.95	6.59	124.67	174	5.72	N
7	**144.7**	**2.35**	278	**27.8**	1.93	**15.2**	**1.46**	**573**	1.32	7.88	296.89	24	0.79	N
9	**10.9**	**1.15**	262	44.7	1.78	3.66	0.61	229	2.92	2.06	128.65	575	18.89	N
10	**23.9**	**4.08**	257	41.6	3.28	**10.9**	**1.19**	298	2.76	3.32	90.85	37	1.22	N
11	<5	**0.818**	386	45.1	1.72	3.68	0.45	184	3.82	2.14	106.98	736	24.18	N
12	**30.4**	**2.56**	273	38.6	3.35	5.23	0.51	265	6.57	1.56	79.10	558	18.33	N
14	<5	**0.865**	336	45.8	2.3	5.2	0.6	215	3.83	2.26	93.48	791	25.99	N
15	**11.4**	**0.556**	**482**	44.6	**1.2**	**9.1**	**0.9**	195	1.33	7.58	162.50	920	30.23	N
16	**10.5**	**0.824**	359	42.6	2	5.52	**0.81**	391	2.47	2.76	195.50	8	0.26	N
17	**26.4**	**2.92**	**1116**	43	**1.3**	4.21	**0.19**	235	6.84	3.24	180.77	203	6.67	N
18	<5	**0.786**	**915**	47.3	2.05	5.84	0.46	184	4.46	2.85	89.76	807	26.51	N
19	**12.6**	**1.04**	357	46.6	2.34	4.59	**0.81**	196	2.89	1.96	83.76	1090	35.81	N
20	**27.5**	**1.06**	389	42.9	2.79	**12.08**	**0.94**	301	2.97	4.33	107.89	767	25.20	N
21	**62.1**	1.02	**881**	40.5	1.5	**11.5**	**0.9**	**614**	1.67	7.67	409.33	709	23.29	N
22	**6.3**	**1.24**	336	35.9	2.1	4.37	0.32	**576**	6.56	2.08	274.29	533	17.51	N
23	<5	0.445	277	48.8	2	4.7	0.7	222	2.86	2.35	111.00	1488	48.89	**Y**
24	<5	0.358	369	48.2	2.8	4.97	0.21	308	13.33	1.78	110.00	796	26.15	N
25	<5	**>5**	330	47.6	1.5	4.2	0.3	228	5.00	2.80	152.00	1286	42.25	N
26	**6.3**	**0.544**	276	39.7	1.99	6.56	0.77	213	2.58	3.30	107.04	226	7.43	N
27	**20.9**	**1.99**	**804**	45.7	2.58	**7.86**	**0.87**	226	2.97	3.05	87.60	196	6.44	N
28	**338.7**	**>5**	**897**	**23.4**	2.62	**28.9**	0.27	**506**	9.70	11.03	193.13	2	0.07	N
29	<5	0.197	**536**	39.5	**1.08**	**9.25**	**1.05**	290	1.03	8.56	268.52	865	28.42	N
30	<5	**0.559**	349	50.8	1.54	4.34	0.35	156	4.40	2.82	101.30	1430	46.98	**Y**
31	**12.9**	**1.82**	385	44.1	1.8	7.32	**1.3**	184	1.38	4.07	102.22	355	11.66	N
32	**95.5**	**4.23**	**508**	34.1	1.7	**9.85**	**0.85**	373	2.00	5.79	219.41	191	6.28	N
33	**22.1**	**1.27**	311	45.2	1.8	6.7	**1.1**	190	1.64	3.72	105.56	1341	45.06	N
35	<5	**0.65**	340	39.6	**0.89**	6.37	0.28	213	3.18	7.16	239.33	25	0.82	N
36	<5	**0.563**	261	42.6	1.91	5.68	0.75	186	2.55	2.97	97.38	766	25.17	N
37	**32.3**	**2.28**	336	34.7	3.15	7.4	0.45	**548**	7.00	2.35	173.97	192	6.31	N
38	**6.2**	**1.51**	**228**	43.5	2.2	4.6	0.6	394	3.67	2.09	179.09	483	15.87	N
39	**6.5**	**2.4**	**500**	44	1.5	4.7	0.6	228	2.50	3.13	152.00	338	11.11	N
40	**15**	0.463	342	47.2	2.2	6.3	0.8	298	2.75	2.86	135.45	539	17.71	N
41	**13.2**	**1.05**	381	47.5	**0.9**	5.5	0.6	262	1.50	6.11	291.11	203	6.67	N
42	**30.9**	**0.886**	**663**	**26.4**	2.07	**9.01**	0.61	167	3.39	4.35	80.68	48	1.58	N
45	<5	**1.83**	407	43.2	**0.6**	3.6	0.4	189	1.50	6.00	315.00	792	26.02	N
46	**10.1**	0.385	405	35.9	**1.26**	3.46	0.25	**96.2**	5.04	2.75	76.35	463	15.21	N
47	<5	**1.82**	384	46	1.9	4.1	0.7	154	2.71	2.16	81.05	1272	41.79	**Y**
48	**128.1**	**2.51**	**215**	36.6	**1.2**	**18.6**	**1.1**	**526**	1.09	15.50	438.33	100	3.29	N
49	**133.1**	**1.75**	361	35.9	2.55	**8.2**	0.5	357	5.10	3.22	140.00	160	5.26	N
50	**17.3**	**1.43**	329	42.6	**1.23**	4.39	0.35	239	3.51	3.57	194.31	139	4.57	N

Supplementary Table 3. (continued)

Pts. No.	CRP (<5mg/L)	D-dimer (<0.5mcg/mL)	LDH 240-480 U/L	Albumin (<34g/L)	ALC (1.5-3.5G/L)	ANC (2-7.5G/L)	AMC (0.2-0.8G/L)	PLT (150-400G/L)	LMR	NLR	PLR	OS (days)	OS (m)	Censored (Y/N)
51	**18**	0.26	306	43.2	2.83	7.24	**0.91**	383	3.11	2.56	135.34	434	14.26	N
52	<5	**>5**	374	45.2	2.66	6.06	0.33	269	8.06	2.28	101.13	133	4.37	N
53	<5	**0.774**	375	47.8	**1.4**	6.7	**0.9**	276	1.56	4.79	197.14	347	11.40	N
54	**10.3**	**3.58**	255	34.1	1.5	3.3	0.6	342	2.50	2.20	228.00	301	9.89	N
55	**44.2**	**>5**	**919**	**33.5**	2.47	2.85	0.48	156	5.15	1.15	63.16	369	12.12	N
56	**9.3**	**2.12**	**830**	**30.5**	2.2	7.09	0.48	**436**	4.58	3.22	198.18	255	8.38	N
57	<5	0.171	330	46.1	1.75	4.36	0.42	267	4.17	2.49	152.57	910	29.90	N
58	<5	**0.648**	**669**	46.6	**1.4**	4.2	**1**	**351**	1.40	3.00	250.71	239	7.85	N
59	<5	**0.707**	336	47.6	2.3	5.2	0.6	161	3.83	2.26	70.00	1131	37.16	**Y**
60	**14.6**	**0.677**	398	45.5	2.09	5.98	0.57	139	3.67	2.86	66.51	104	3.42	N
61	**10.3**	**0.731**	**235**	44.7	2.1	4.8	**0.9**	257	2.33	2.29	122.38	549	18.04	N
62	**19.8**	**1.08**	321	40.4	2.1	**8**	0.7	301	3.00	3.81	143.33	596	19.58	N
64	**27.2**	**>5**	401	**29.8**	**3.8**	3.7	0.7	283	5.43	0.97	74.47	554	18.20	N
65	**10.1**	**1.62**	327	41.4	1.9	7.2	0.9	344	2.11	3.79	181.05	693	22.77	N
67	**17.5**	**1.98**	**1146**	41.1	1.59	6.34	**1.15**	257	1.38	3.99	161.64	103	3.38	N
68	**16.3**	**3.1**	388	46.8	3.37	**8.65**	0.54	232	6.24	2.57	68.84	98	3.22	N
69	**6.5**	**0.571**	307	43.3	**1.31**	2.5	0.24	306	5.46	1.91	233.59	675	22.18	N
70	**54.3**	**4.4**	**1178**	**37.7**	**1.1**	7.5	0.6	311	1.83	6.82	282.73	199	6.54	N
72	**26.3**	**1.69**	**910**	46.6	2.4	**13.7**	**1.4**	**355**	1.71	5.71	147.92	145	4.76	N
73	**67.9**	0.44	459	43.8	1.5	**17.5**	**1.5**	**455**	1.00	11.67	303.33	80	2.63	N
75	<5	0.17	381	49.6	1.9	6.5	**1.2**	224	1.58	3.42	117.89	963	31.64	**Y**
76	**19.2**	**1.44**	**1788**	43.3	3.13	4.19	0.51	314.1	6.14	1.34	100.35	272	8.94	N
77	**55.4**	**1.02**	**967**	**33.9**	1.53	**17.8**	0.62	**573**	2.47	11.63	374.51	11	0.36	N
78	<5	0.1	261	37.1	1.73	3.96	0.36	253	4.81	2.29	146.24	898	29.50	N
79	**13.7**	**5**	467	41.7	**0.6**	**10.6**	0.52	243	1.15	17.67	405.00	23	0.76	N
80	<5	0.42	**616**	47.1	1.9	4.7	0.4	291	4.75	2.47	153.16	234	7.69	N
81	**60**	**1.49**	**488**	39.5	**1**	7.5	0.6	356	1.67	7.50	356.00	138	4.53	N
84	**6.4**	**0.96**	**992**	44.8	**1.35**	**12.1**	0.57	199	2.37	8.96	147.41	545	17.91	N
85	<5	**0.86**	274	43.4	1.91	6.23	0.56	250.9	3.41	3.26	131.36	731	24.02	**Y**
87	<5	**1.44**	314	41.6	**1.2**	3.2	0.5	228	2.40	2.67	190.00	230	7.56	N
88	**13.7**	**0.97**	327	45.2	**1.3**	**7.7**	0.7	332	1.86	5.92	255.38	411	13.50	N

Supplementary Table 4. Description of the therapy administered

Pts. No.	Therapy
1	RT
2	3xFOLFIRINOX
4	9xTXT, 3xFOLFIRINOX
5	4xGEM+CDDP
6	3xTeysuno+CDDP, 2xCAPE
7	1xCAPE
9	4xEC, 1xTAX
10	1xFOLFIRINOX
11	8xBEV+FOLFOX, 4xBEV+DeGramont, 6xBEV+FOLFOX, 4xBEV+DeGramont, 8xBEV+FOLFIRI, 3xBEV+IRI, 2xmono IRI+EVE
12	7xTXT
14	10xTXT+CAPE
15	1xTXT, abirateron, enzalutamid
16	1xTPF
17	1xTXT+CDDP
18	2xFOLFOX, 5xPAN+FOLFOX, 1xFOLFOX, 2xPAN+FOLFOX, 5xBEV+FOLFIRI, 7xBEV+Degramont, 7xFOLFIRI, 5xPAN+MEKi+BRAFi, regorafenib
19	7xTPF, 2xCDDP+5FU
20	1xFOLFOX, 11xCAPEOX, palliative resection, 4xCAPE, palliative chemo-RT, 6xCAPIRI
21	14xGEM+CDDP, 3xmonoGEM
22	10xTEY+CDDP
23	3xPALBO+FULV, monoFULV, TAM,
24	1xTPF, KRT
25	4xPEM+CBP
26	Palliative RT
27	1xFOLFOX, 4xPAN+FOLFOX, 3xBEV+CAPIRI
28	1xGEM+CDDP
29	13xTOPO
30	1xFOLFIRI, 2xBEV+FOLFIRI, CLS+HIPEC, 1xCAPEOX, 3xCAPE, left inguinal RT, trifluridin+tipiracil, left pelvic lymphnode SBRT, trifluridin+tipiracil
31	6xCET+CDDP+5FU, 2xNIVO
32	6xTAX+CBP
33	3xTPF
35	1xTPF
36	6xTPF, RT, 3xCET+CDDP+5FU
37	12xFOLFIRINOX
38	2xFOLFOX, 3xBEV+FOLFOX, 17xBEV+FOLFOXIRI
39	PALBO+LETRO
40	4xGEM+CBP, 7xTXT
41	5xPAN+FOLFOX
42	4xBEV+FOLFIRI
45	3xFOLFIRI, 10xBEV+FOLFIRI+6xEVE, DEB-TACE, trifluridin+tipiracil, monoCAPE
46	7xBEV+CAPIRI
47	ENZA
48	2xATEZO
49	4xTEY+CDDP
50	8xFOLFIRINOX

Supplementary Table 4. (continued)

Pts. No.	Therapy
51	10xCET+CDDP+5FU
52	9xFOLFIRINOX
53	3xBEV+TAX+CBP, RT,
54	6xBEV+FOLFOX, 3xFOLFIRI, 6xBEV+FOLFIRI
55	AI, PALBO, 3xEC, 1xTXT+CAPE, 3xTXT
56	2xTAX+CBP, 4xmonoCBP
57	4xTPF, RT
58	4xTPF, followed by 4xCET+CDDP+5FU (for pulm.met.)
59	5xTXT+HER+PER, followed by HER+PER+FULV
60	3xGEM+CBP
61	4xTPF, RT, 2xCDDP+5FU, 2xCET+CDDP+5FU
62	8xBEV+FOLFOX, 8xBEV+DeGramont, 13xBEV+FOLFIRI
64	No consent to surgery, RT, TEY+CDDP
65	8xBEV+FOLFOX, 2xBEV+DeGramont, no consent to further therapy
67	6xBEV+FOLFOX
68	2xGEM+CBP, 2xTXT+CBP
69	8xBEV+FOLFOX, 2xBEV+DeGramont, 15xPAN+FOLFIRI
70	1xFOLFOX, 4xPAN+FOLFOX, 3xBEV+FOLFIRI
72	3xFOLFIRINOX, 1xGEM
73	1XGEM
75	4xCBP+ETO, 15xTXT
76	4xEC, 5xTAX+CBP
77	1xTPF
78	11xFOLFIRINOX, 10xnabTAX+GEM
79	1XGEM
80	4xBEV+TAX+CBP, 2xCrizotinib
81	6xCDDP+ETO, 1xTXT+HER
84	3xCET+CDDP+5FU, RT, NIVO
85	RIBO+LETRO
87	7xFOLFIRINOX
88	6xCDDP+ETO

Supplementary Table 5. Description of comorbidities

Pts. No.	Comorbidities
1	left leg varicectomy, left shoulder dislocation, smoking
2	varicella, tonsillectomy, left 5th finger fracture
4	-
5	anxiety depression, COPD
6	hypertension, varicectomy L.II. Vertebral fracture
7	left tibial, left humeral fracture, glaucoma, hypertension, gout, 2TDM, DCM, AF, CRF st. III.
9	uterinal abrasion, uteral suture, right breast lumpectomy, SLNB (Hist.: IDC, Gr. II, pT1c,pN0,M0, ER 60%, PR 40%), adjuvant irradiation, tamoxifen, (16ys ago) left lateral ankle fracture
10	varicella
11	varicella, pertussis, mumps, rubeola, morbilli, childhood pneumonia, right hip TEP, AF, mitral endocarditis
12	cholecystectomy, left hip TEP, nephrolithiasis, right wrist fracture, right 5th finger distal phalanx amputation, hypertension, PSVT, ablation of the slow pathway, prostate cancer (for 8 ys)
14	cervical hernia, right ovarectomy, hypertension , IHD, chr. bronchitis, emphysema pulm., left breast excision and ABD (hist. IDC), 6xTAX+EPI, RT, ET, (8ys ago), left mastectomy, 6xTAX+CBP (2ys ago)
15	hypertension, AF, prostate carcinoma
16	-
17	colon ACC resection, adj. Mayo th. (12ys ago)
18	tonsillectomy, chest surgery (pectus carinatum), testicular cyst surgery, right IV. left III. Finger distal phalanx injury, left inguinal herniotomy
19	COPD
20	-
21	rubeola, varicella, tonsillectomy, thyroid surgery (goiter), appendectomy, depression, COPD
22	tonsillectomy, meniscus injury
23	COPD
24	rib fracture, clavicule fracture
25	pulmonary ACC, 4xTAX+CBP, Chemo-RT (3ys ago)
26	AF, hysterectomy, hypertension, glaucoma, cataracta
27	appendectomy, myocardial inf., gout, hypertension
28	COPD, rectal adenocc., 8xFOLFOX, 4xDeGramont (3ys ago)
29	hypertension, AF, COPD, myocardial inf., CF
30	GERD, sigmoid ACC resection, 12xFOLFOX (4ys ago)
31	tonsillectomy, aethyl chr., smoking, asthma bronchiale, L-IV discectomia, lichen planus, prostatitis chr., GERD, gastritis, ulcus duodeni, hyperlipidaemia
32	asthma bronchiale, strumectomy
33	inguinal herniotomy, hypertension, stroke
35	COPD
36	hepatic cirrhosis
37	varicella, mumps, rubeola, scarlatina, morbilli, hysterectomia (pp. myoma uteri), strangular ileus (adhesions), gastric ulcer, gastric perforation
38	varicella, left humeral fracture, appendectomy
39	-
40	COPD, hypertension, myocardial inf.
41	tibial fracture, varicectomy, hypertension
42	COPD, aethylism
45	hypertension, 2TDM, hypercholesterinaemia, left nephrectomy, rectal ACC resection, 6xFOLFOX, Chemo-RT (1 y ago)
46	varicella, Caesarean section, appendectomy, 2TDM, LC, hyperthyreosis
47	appendectomy, hypertension, PSVT , hypothyreosis, CRF, prostate carcinoma (ADT, EBRT) (3ys ago)
48	COPD
49	COPD, aethylismus
50	gout, dysphonia, appendectomy, hypertension

Supplementary Table 5. (continued)

Pts. No.	Comorbidities
51	gastric ulcer, hypertension
52	surgery of right nephrolithiasis, hypertension
53	varicella, tonsillectomy, cholecystectomy, left breast cyst excision, wrist fracture, hypertension
54	IHD
55	varicella, rubeola, tonsillectomy, hypertension, anxiety, COPD
56	-
57	aethylismus
58	appendectomy, COPD, aethylismus
59	varicella, mumps, rubeola, tonsillectomy, bilateral salivary gland inflammation, left salivary gland surgery, periferial facial paresis, vocal chord polyp surgery, cholecystectomy, hypertension
60	2TDM, hypertension, COPD, CRF
61	varicella, tonsillectomy, discus hernia (L.IV-V) surgery, aethylismus, left femoral stenosis, right-left cross over bypass graft, left lumbar sympathectomy
62	COPD
64	hiatus hernia, esophegeal diverticulum
65	hypertension
67	left inguinal herniotomy, right orchiectomy, LC, CABG, right hip fracture, L-V vertebral fracture, TURP
68	ESWL, PCNL, nodular goiter surgery, left nephrectomy (renal cancer) (2ys ago)
69	tonsillectomy, left VI rib fracture, left inguinal herniotomy, hypertension
70	hypertension
72	varicella, rubeola, tonsillectomy, hypertension, right carotid stenosis
73	tonsillectomy, strumectomy, COPD
75	varicella, mumps, gastric ulcer perforation, hypertension, polypectomy, AF, CF
76	tonsillectomy, hyperthyreosis, hypertension, TNBC (PS 6xTAX+CBP, mastectomy, RT) (4ys ago)
77	tonsillectomy, renal abscessus surgery
78	varicella
79	cholecystectomy, hysterectomy, hypertension, sleep apnoe sy., AF
80	varicella, hysterectomy, left ovarectomy, goiter surgery, left hallux valgus surgery, left lateral ankle fracture
81	left elbow fracture, tonsillectomy, Caesarean section
84	left lateral ankle fracture, gastric perforation
85	hypertension
87	bilateral pneumonia, hypertension, right hip TEP, left wrist fracture
88	varicella, scarlatina, pertussis, tonsillectomy, left cataracta surgery

Supplementary Table 6. Data of the six cenzored patients

Pts. No.	CRP (mg/L)	albumin (g/L)	NLR	OS (months)
23	<5	48.8	2.35	48.89
30	<5	50.8	2.82	46.98
47	<5	46	2.16	41.79
59	<5	47.6	2.26	37.16
75	<5	49.6	3.42	31.64
85	<5	43.4	3.26	24.02

Supplementary Table 7. Distribution of the patients after stratification into four prognostic groups*

	Group 1	Group 2			Group 3			Group 4
CRP (mg/L)	≤ 30.65	**> 30.65**	≤ 30.65	≤ 30.65	**> 30.65**	**> 30.65**	≤ 30.65	**> 30.65**
albumin (g/L)	> 44.35	> 44.35	**≤ 44.35**	> 44.35	**≤ 44.35**	> 44.35	**≤ 44.35**	**≤ 44.35**
PLR	≤ 168.20	≤ 168.20	≤ 168.20	**> 168.20**	≤ 168.20	**> 168.20**	**> 168.20**	**> 168.20**
Pts. No.	9; 11; 14; 15; 18; 19; 23; 24; 25; 27; 30; 33; 40; 47; 52; 57; 59; 60; 61; 68; 72; 75; 80; 84;		4; 5; 10; 12; 20; 26; 31; 36; 39; 46; 51; 62; 64; 67; 76; 78; 85;	41; 53; 58; 88;	1; 2; 6; 42; 49; 55;		16; 17; 22; 29; 35; 38; 45; 50; 54; 56; 65; 69; 79; 87;	7; 21; 28; 32; 37; 48; 70; 73; 77; 81;
Total	24	0	17	4	6	0	14	10

* = no patient was classified to subgroup with either single increased CRP or doublet increased CRP and PLR

Supplementary Table 8. Summary of the patient characterstics of the Group 1 (n=24)

No.	Diagnosis	Histology	Stage	Metastasis in St. IV.	Therapeutic response for 1st line th. (m)	OS (m)
9	Rec.BC	non specified type	III	-	14.00	18.89
11	mCRC	adenocarcinoma	IV	liver	12.58	24.18
14	mBC	non specified type	IV	neck lymphnode	5.26	25.99
15	mCRPC	adenocarcinoma	IV	bone, pulmonary	4.04	30.23
18	mCRC	adenocarcinoma	IV	liver	5.65	26.51
19	RecHNSCC	squamous cell cc.	III	-	19.97	35.81
23	mBC	non specified type	IV	bone, pulmonary	4.34	48.89 (cens.)
24	LA-HNSCC	squamous cell cc.	III	-	26.15	26.15
25	mNSCLC	adenocarcinoma	IV	bone	6.93	42.25
27	mCRC	adenocarcinoma	IV	peritoneal c., liver	2.76	6.44
30	mCRC	adenocarcinoma	IV	peritoneal	10.12	46.98 (cens.)
33	LA-HNSCC	squamous cell cc.	III	-	1.94	45.06
40	LA-NSCLC	squamous cell cc.	III	-	8.51	17.71
47	mCRPC	adenocarcinoma	IV	bone	41.79 (cens.)	41.79 (cens.)
52	mPC	adenocarcinoma	IV	liver	2.73	4.37
57	LA-HNSCC	squamous cell cc.	III	-	29.90	29.9
59	mBC	non specified type	IV	bone	37.16 (cens.)	37.16 (cens.)
60	LA-NSCLC	adenocarcinoma	III	-	3.42	3.42
61	LA-HNSCC	squamous cell cc.	III	-	9.99	18.04
68	mNSCLC	adenocarcinoma	IV	pulmonary, bone	2.66	3.22
72	mPC	adenocarcinoma	IV	liver	2.00	4.76
75	ED-SCLC	cc. microcell.	III	-	3.68	31.64 (cens.)
80	LA-NSCLC	adenocarcioma	III	-	2.53	7.69
84	mHNSCC	squamous cell c.	IV	bone	17.91	17.91

Supplementary Table 9. Summary of the patient characterstics of the Group 2 (n=21)

No.	Diagnosis	Histology	St.	Metastasis in St. IV.	Therapeutic response for 1st line th. (m)	OS (m)
4	mHSPC*	adenocarcinoma	IV	bone	4.79	11.43
5	mNSCLC	adenocarcinoma	IV	pulmonary, liver	16.92	25.07
10	mPC	adenocarcinoma	IV	pulmonary, brain	1.21	1.22
12	mCRPC	adenocarcinoma	IV	bone, liver, pulm.	5.16	18.33
20	LA-CRC	adenocarcinoma	III	-	15.28	25.2
26	LA-HNSCC	squamous cell cc.	III	-	6.93	7.43
31	mHNSCC	squamous cell c.	IV	pulmonary	6.24	11.66
36	mHNSCC	squamous cell cc.	IV	adrenal	18.66	25.17
39	mBC	non specified type	IV	bone, pulmonary	10.41	11.11
41	mCRC	adenocarcinoma	IV	liver	5.68	6.67
46	mCRC	adenocarcinoma	IV	liver	11.69	15.21
51	mHNSCC	squamous cell cc.	IV	pulmonary	8.97	14.26
53	LA-NSCLC	adenocarcinoma	III	-	5.09	11.4
58	LA-HNSCC	squamous cell cc.	III	-	3.88	7.85
62	mCRC	adenocarcinoma	IV	pulmonary	12.19	19.58
64	LA-GC	adenocarcinoma	III	-	No consent to th.	18.20
67	mCRC	adenocarcinoma	IV	pulmonary, liver	3.38	3.38
76	Rec.BC	non specified type	III	-	7.36	8.94
78	LA-PC	adenocarcinoma	III	-	10.45	29.5
85	mBC	non specified type	IV	bone	24.02 (cens.)	24.02 (cens.)
88	ED-SCLC & HNSCC	cc. microcell. & squamous cell c.	III	-	13.50	13.5

Supplementary Table 10. Summary of the patient characterstics of the Group 3 (n=20)

No.	Diagnosis	Histology	St.	Metastasis in St. IV.	Therapeutic response for 1st line th. (m)	OS (m)
1	LA-NSCLC	adenocarcinoma	III	-	23.62	23.62
2	mPC	adenocarcinoma	IV	pulmonary	2.76	4.07
6	mGC	adenocarcinoma	IV	liver	2.50	5.72
16	LA-HNSCC	squamous cell cc.	III	-	0.26	0.26
17	mNSCLC	adenocarcinoma	IV	pleural carcinosis	5.49	6.67
22	Rec.GC	adenocarcinoma	III	abd. lymphnode	16.49	17.51
29	mOC	adenocarcinoma	IV	pulmonary	28.29	28.42
35	LA-HNSCC	squamous cell cc.	III	-	0.82	0.82
38	mCRC	adenocarcinoma	IV	liver	15.14	15.87
42	mCRC	adenocarcinoma	IV	pulmonary, liver	1.58	1.58
45	mCRC	adenocarcinoma	IV	pulmonary, liver	6.93	26.02
49	mGC	adenocarcinoma	IV	perit. c., bone	3.68	5.26
50	mPC	adenocarcinoma	IV	liver	4.07	4.57
54	mCRC	adenocarcinoma	IV	liver	3.35	9.89
55	mBC	non specified type	IV	peritoneal c.	3.42	12.12
56	LA-OC	adenocarcinoma	III	-	4.83	8.38
65	mCRC	adenocarcinoma	IV	peritoneal c., liver	10.55	22.77
69	mCRC	adenocarcinoma	IV	liver	8.87	22.18
79	mPC	adenocarcinoma	IV	liver	0.76	0.76
87	mCholecyst	adenocarcinoma	IV	liver	3.78	7.56

Supplementary Table 11. Summary of the patient characterstics of the Group 4 (n=10)

No.	Diagnosis	Histology	St.	Metastasis in St. IV.	Therapeutic response for 1st line th. (m)	OS (m)
7	mGC	adenocarcinoma	IV	peritoneal c.	0.79	0.79
21	mNSCLC	squamous cell c.	IV	bone	19.32	23.29
28	mNSCLC	adenocarcinoma	IV	bone	0.07	0.07
32	mOC	adenocarcinoma	IV	pulmonary	6.28	6.28
37	mPC	adenocarcinoma	IV	liver	5.68	6.31
48	mBladder	transitiocell. c.	IV	pulmonary	3.29	3.29
70	LA-CRC	adenocarcinoma	III	-	4.99	6.54
73	mNSCLC	squamous cell c.	IV	pulmonary, bone	2.63	2.63
77	LA-HNSCC	squamous cell c.	III	-	0.36	0.36
81	mBC	neuroendocrine c.	IV	mediastinal, bone	3.68	4.53
